# Non-linear Associations Between Visceral Adiposity Index and Cardiovascular and Cerebrovascular Diseases: Results From the NHANES (1999–2018)

**DOI:** 10.3389/fcvm.2022.908020

**Published:** 2022-06-24

**Authors:** Yangchang Zhang, Qiang He, Weihao Zhang, Yang Xiong, Shisi Shen, Jialu Yang, Mengliang Ye

**Affiliations:** ^1^School of Public Health and Management, Chongqing Medical University, Chongqing, China; ^2^Research Center for Medicine and Social Development, Chongqing Medical University, Chongqing, China; ^3^The Innovation Center for Social Risk Governance in Health, Chongqing Medical University, Chongqing, China; ^4^Research Center for Public Health Security, Chongqing Medical University, Chongqing, China; ^5^Department of Neurosurgery, West China Hospital, Sichuan University, Chengdu, China; ^6^Department of Neurosurgery, The Affiliated Hospital of Guizhou Medical University, Guizhou Medical University, Guiyang, China; ^7^West China Hospital, Sichuan University, Chengdu, China; ^8^The First School of Clinical Medicine, Chongqing Medical University, Chongqing, China

**Keywords:** visceral adiposity index, angina, heart attack, stroke, hypertension, coronary heart disease

## Abstract

**Objective:**

To investigate associations between visceral adiposity index (VAI) and cardiovascular and cerebrovascular diseases (CCDs) in the American population from 1999 to 2018.

**Methods:**

Data from the National Health and Nutrition Examination Survey (1998–2018) were analyzed in this study. Specifically, VAI scores were calculated using sex-specific equations that incorporate body mass index, waist circumference (WC), high-density lipoprotein (HDL), triglycerides (TG), and cholesterol. Weighted logistic regression analysis was conducted to assess the relationship between VAI tertile and increased risk of CCDs. Restricted cubic splines were used to evaluate the non-linear relationship between VAI and CCDs, such as heart failure, angina, heart attack, stroke, hypertension, and coronary heart disease. Sensitivity analysis was conducted, using VAI quartiles as independent variables.

**Results:**

A total of 22,622 subjects aged over 20 years were included. In the fully adjusted model after controlling for covariates, the third VAI tertile was more strongly associated with CCDs than the first VAI tertile, with odds ratio (OR) and 95% confidence interval (95% CI) values for angina of 2.86, 1.68–4.85; heart attack, 1.75, 1.14–2.69; stroke, 2.01, 1.23–3.26; hypertension, 2.28, 1.86–2.78; and coronary heart disease, 1.78, 1.32–2.41; but there was no significant association with heart failure (*p* > 0.05). Restricted cubic splines revealed parabolic relationships between VAI score and angina (*p* for non-linear = 0.03), coronary heart disease (*p* for non-linear = 0.01), and hypertension (*p* for non-linear < 0.001). Sensitivity analysis indicated that the fourth VAI quartile was more strongly associated with an increased risk of angina (OR = 2.92, 95% CI, 1.49–5.69), hypertension (OR = 2.37, 95% CI, 1.90–2.97), heart attack (OR = 1.77, 95% CI, 1.09–2.88), and coronary heart disease (OR = 1.89, 95% CI, 1.24–2.86) than the first VAI quartile. VAI had superior predictive power for prevalent CCDs than other independent indicators (*p* < 0.05).

**Conclusion:**

Visceral adiposity index score is positively correlated with angina, heart attack, stroke, hypertension, and coronary heart disease, but not heart failure, and the relationships between VAI score and angina, hypertension, and coronary heart disease are non-linear.

## Introduction

Cardiovascular and cerebrovascular diseases (CCDs) are major causes of death in the United States of America (USA), and morbidity and mortality rates attributable to CCDs are increasing. Approximately 659,000 people in the USA die from heart disease each year, and the cost associated with these diseases was up to $363 billion each year in 2016 and 2017 (1). Similarly, the burden of cerebrovascular disease has emerged over the past decade (1). Around 795,000 initial or recurrent acute strokes were reported each year, with an estimated annual financial cost of $17.9 billion that includes medicines, healthcare service expenses, and economic losses due to death between 2012 and 2013 (2). Researchers have identified many factors, which influence the development of CCDs, such as genetics, lifestyle, dietary behavior, age, secondary behavior, and cardiometabolic diseases (3).

The close association among visceral adiposity, inflammation, and cardiometabolic disease has been widely discussed, and there is a general view that visceral obesity is a sign of ectopic fat infiltration and dysfunctional adipose tissue, with high visceral obesity thus more likely to be associated with chronic inflammation or cardiometabolic syndrome (4, 5). Visceral obesity can be assessed by magnetic resonance imaging (MRI) or computed tomography (CT) scan, and the results were transformed to a scale of 1–59, where scores below 13 are considered to indicate good health, while those between 13 and 59 are indicative of poor condition (6).

Although both MRI and CT are considered gold standard methods and have good validity for clinical diagnosis of body composition because of their sensitivity and specificity, these approaches may exclude participants with lower income, and in large sample populations, the high cost of MRI or CT represents a burden on participants, making them inappropriate for use in screening (7). Alternatively, the visceral adiposity index (VAI) has been validated as a reliable indicator of adipose distribution and function, which indirectly reflects individual cardiometabolic risk (8). The sex-specific VAI is an empirical-mathematical model that consists of anthropometric [body mass index (BMI) and waist circumference (WC)] and blood biomarker [high-density lipoprotein (HDL) and triglycerides (TG)] data, forming two linear equations that can be applied to estimate visceral adiposity (8) (Table 1).

**TABLE 1:** Sex-specific equations for calculation of the visceral adiposity index.

Sex	Visceral Adiposity Index (VAI) equation
Male	VAI = [WC/39.68 + (1.88 × BMI)] × (TG/1.03) × (1.31/HDL)
Female	VAI = [WC/36.58 + (1.89 × BMI)] × (TG/0.81) × (1.52/HDL)

*WC, waist circumference; BMI, body mass index; TG, triglycerides; HDL, high density lipoprotein.*

Associations between cardiovascular disease (CVD) and VAI have been reported previously. The ATTICA study, a large-sample longitudinal study in Greece, reported that VAI is independently associated with increased 10-year CVD risk (9). Another prospective study of 464 prevalent hemodialysis patients also suggested that VAI had superior power for identifying CVD than WC and waist-to-height ratio (10). For CVD subtypes, a cross-sectional study of 460 Hungarians indicated that VAI was a significant predictor for coronary calcification (4), while a prospective study that included 780 Chinese subjects found that VAI at baseline was an independent risk indicator and early marker of the incident in hypertension (11); however, investigations of the association between VAI and cerebrovascular diseases are scarce.

There are limited data on the associations between VAI and CCDs in the USA. Moreover, the non-linearity of relationships between VAI and CCDs warrants investigation in a large-sample survey. The purpose of this study was to examine the associations between VAI and CCDs over an 18-year period in the USA that included an analysis of all possible CCDs (i.e., heart failure, heart attack, coronary heart disease, hypertension, angina, and stroke). In addition, we applied the spline regression statistical method to examine the non-linear nature of the relationships.

## Materials and Methods

### Ethical Approval

Ethical review and approval were waived for this study since all the data from the National Health and Nutrition Examination Survey (NHANES) are publicly accessible. The NHANES was approved by the National Centre for Health Statistics Institutional Ethics Review Board, and all the subjects agreed to complete the survey and provided written consent. The datasets used in this work were released on the NHANES website.^1^

### Survey Design and Study Population

The NHANES is an ongoing nationally representative health examination and nutritional status survey of adults and children in the USA, conducted in a series of cross-sectional waves from 1998 to 2018, which includes three components: questionnaire interviews, physical tests, and laboratory examinations (12). Questionnaire interviews were carried out in each subject’s home, and physical tests and laboratory examinations were then completed in mobile examination centers. Detailed information about the study design and quality control has been released in a previous paper (12).

**FIGURE 1:**
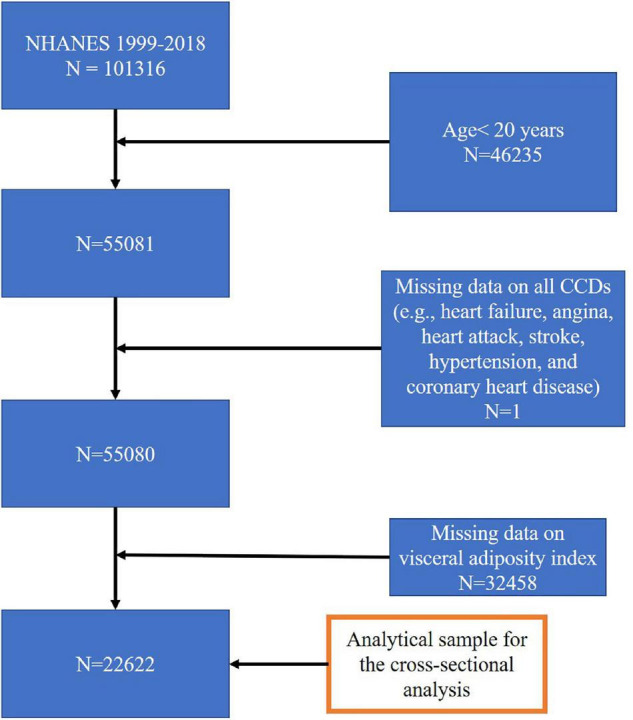
Sample selection process flow chart.

In the present study, we extracted the data from ten survey cycles (1999–2000, 2001–2002, 2003–2004, 2005–2006, 2007–2008, 2009–2010, 2011–2012, 2013–2014, 2015–2016, and 2017–2018). In total, there were 101,316 subjects in the pooled cycles. Subjects with missing values for all CVDs and/or VAI, or aged below 20 years, were excluded from the sample pool. The final sample for multiple cross-sectional analysis comprised of 22,622 subjects. The study flow chart is presented in Figure 1.

### Outcome Variable: Cardiovascular and Cerebrovascular Diseases

Six types of a CCD diagnosed by a doctor or a health professional were defined as outcomes, such as heart failure, angina, heart attack, stroke, hypertension, and coronary heart disease. The items, “ever told has congestive heart failure,” “ever told you had angina/angina pectoris,” and “ever told you had a heart attack” were used to identify participants with heart failure, angina, and heart attack, respectively; participants were defined as having coronary heart disease when one or more of these diseases were confirmed, combined with the specific item “ever told you had coronary heart disease.” The definition of hypertension had three parts: first, the questionnaire item, “ever told you had high blood pressure” was self-reported hypertension; second, average systolic pressure higher than 140 mm/Hg measured four times, or average diastolic pressure higher than 90 mm/Hg measured four times; third, the item “taking a prescription for hypertension” was also adopted to identify participants with hypertension.

### Exposure Variable: Visceral Adiposity Index

In the NHANES, VAI was calculated using two separate equations for men and women. The equation for men was VAI = (WC/[39.68 + 1.88 × BMI)] × (TG/1.03) × (1.31/HDL) and the formula for women was VAI = [WC/(36.58 + 1.89 × BMI)] × (TG/0.81) × (1.52/HDL), where WC is expressed in cm and TG and HDL as mmol/L (Table 1). A higher VAI score indicates greater estimated visceral adiposity, which is associated with a higher risk of CCDs.

### Covariates

Some variables were included as covariates, to correct for false correlations, such as age, sex, ethnicity (Mexican American, black, white, and other race), and educational level (lower than high school, high school graduate or equivalent degree, and college graduate or above). Annual family income was divided into two classes: ≤ 20,000, and > 20,000 USD. Smoking status was classified as never smoking, former smoking, and current smoking. Diabetes was identified by self-reported diabetes, use of diabetes medication or insulin, glycohemoglobin or hemoglobin A1c (HbA1c) (%) > 6.5, and fasting glucose (mmol/l) ≥ 7.0.

### Statistical Analysis

Visceral adiposity index was grouped into tertiles from the lowest (first tertile, Q1) to the highest (third tertile, Q3). In general, samples were characterized by calculating means and standard deviations (SDs) for continuous variables, and the number of cells and proportions for categorical variables. Inferential statistics for comparison of individual characteristics between VAI subgroups were determined using the Kruskal–Wallis rank-sum test for data with skewed distribution, one-way ANOVA for data with normal distribution, and chi-square test for categorical data. A ten-cycle sample weight (1999–2018) was used to account for oversampling. Corresponding primary sample unit, sample strata, and sample center were also defined to account for complex multistage probability sampling, based on tutorials on the NHANES website. Additionally, Taylor-linearized variance estimation was adopted to acquire variance estimators of calibration estimators of totals and non-linear finite population parameters. This variance estimation method is generally applicable to complex survey designs. Three multiple logistic regression analysis models were used to assess associations of VAI score tertiles with heart failure, angina, heart attack, stroke, hypertension, and coronary vascular disease. Model 1 was adjusted for age and sex; Model 2 was further adjusted for ethnicity, income, and educational level; and Model 3 was further adjusted for smoking and diabetes. The predictive power of VAI and each independent indicator (e.g., BMI, WC, TG, and HDL) was assessed using receiver-operating characteristic (ROC) curve analysis. Differences in the area under the ROC curve (AUC) values were compared between VAI and other indicators. Subgroup analyses of data stratified by sex and ethnicity were conducted to determine potential interaction effects. Restricted cubic spline regression, controlling for all covariates, was conducted with four knots (at 25, 50, 75, and 95) to investigate the non-liner relationship between exposure variables and outcomes. In sensitivity analysis, VAI scores were treated as quartiles in the full logistic model to investigate their associations with CCDs. Multinomial logistic regression was used to estimate associations between VAI and multiple CCD hits. The significant threshold was defined as *p* < 0.05. STATA software (version 16.1, Stata Corporation) and *R* studio (version 4.1.2) were used to perform all analyses. The “nhanesR” package was used to extract NHANES datasets.

## Results

### Sample Characteristics

The characteristics of samples across VAI tertiles between 1999 and 2018 are shown in Table 1. A total of 22,622 participants were included in the final analysis, and the unweighted mean VAI values were 0.61 ± 0.19, 1.32 ± 0.26, and 3.51 ± 1.88 for the first, second, and third tertiles, respectively. The mean age and proportions of participants who were women, who were white, had high income, had a high education level, had obesity, were ex-smokers and current smokers, and had diabetes and prediabetes were increased with higher VAI tertile. Further, the percentages of participants with heart failure (1.9 and 4.3%), angina (1.4 and 3.7%), heart attack (2.9 and 5.4%), stroke (2.7 and 4.3%) were higher in participants in the third than the first tertile, while hypertension (26.6 and 42.4%) and coronary heart disease (6.9 and 12.4%) were markedly higher in the first when compared with the third VAI tertile.

### Associations of Visceral Adiposity Index Tertiles With Cardiovascular and Cerebrovascular Diseases

In the full model, a higher VAI score was positively associated with heart failure. After controlling for age, sex, ethnicity, income, educational level, smoking status, prediabetes, and diabetes, the odds ratio (OR) values for heart failure for second and third tertile VAI scores were 1.57 [95% confidence interval (CI), 0.86–2.85] and 1.89 (95% CI, 0.94–3.80), respectively, relative to the first tertile (reference); while those for angina, heart attack, stroke, hypertension, and coronary heart disease were 1.80 (95% CI, 0.97–3.36) and 2.86 (95% CI, 1.68–4.85); 1.34 (95% CI, 0.86–2.07) and 1.75 (95% CI, 1.14–2.69); 1.78 (95% CI, 1.03–3.06) and 2.01 (95% CI, 1.23–3.26); 1.56 (95% CI, 1.30–1.87) and 2.28 (95% CI, 1.86–2.78); and 1.42 (95% CI, 1.06–1.91) and 1.78 (95% CI, 1.32–2.40), respectively, (Table 2). The VAI had better predictive power for prevalent CCDs than other independent indicators, as indicated by the AUC value (*p* < 0.001; Supplementary Table 1). The AUC (95% CI) values of VAI for heart failure, angina, heart attack, stroke, hypertension, and coronary heart disease were 0.58 (0.56–0.60), 0.58 (0.56–0.60), 0.56 (0.54–0.57), 0.54 (0.52–0.56), 0.54 (0.52–0.56), and 0.56 (0.55–0.57).

**TABLE 2:** Characteristics of NHANES samples (1999–2018).

Variable		VAI T1	VAI T2	VAI T3	*P*
			
		*N* = 5803	*N* = 7751	*N* = 9068	
Age (years)		46.2 (18.2)	49.8 (18.2)	51.4 (17.3)	< 0.001
Sex	Female	2782 (47.9%)	4050 (52.3%)	4853 (53.5%)	< 0.001
	Male	3021 (52.1%)	3701 (47.7%)	4215 (46.5%)	
Ethnicity	Mexican American	704 (12.1%)	1304 (16.8%)	2065 (22.8%)	< 0.001
	Non-Hispanic Black	1781 (30.7%)	1614 (20.8%)	1059 (11.7%)	
	Non-Hispanic White	2293 (39.5%)	3450 (44.5%)	4369 (48.2%)	
	Other	1025 (17.7%)	1383 (17.8%)	1575 (17.4%)	
Income	≤ 20000 USD	413 (25.2%)	737 (27.8%)	1213 (32.6%)	< 0.001
	> 20000 USD	1227 (74.8%)	1911 (72.2%)	2513 (67.4%)	
Education	< High school	1211 (20.9%)	1954 (25.2%)	2941 (32.5%)	< 0.001
	High school	1225 (21.1%)	1793 (23.2%)	2164 (23.9%)	
	> High school	3362 (58.0%)	3998 (51.6%)	3947 (43.6%)	
BMI	Underweight	579 (10.0%)	338 (4.4%)	119 (1.3%)	< 0.001
	Normal	2395 (41.3%)	2031 (26.2%)	1343 (14.8%)	
	Overweight	1690 (29.1%)	2780 (35.9%)	3255 (35.9%)	
	Obese	1139 (19.6%)	2602 (33.6%)	4351 (48.0%)	
Smoking	Former	1308 (22.6%)	1947 (25.1%)	2464 (27.2%)	< 0.001
	Never	3423 (59.1%)	4244 (54.8%)	4514 (49.8%)	
	Now	1064 (18.4%)	1553 (20.1%)	2085 (23.0%)	
Diabetes	Diabetes	559 (9.8%)	1262 (16.7%)	2422 (27.9%)	< 0.001
	Prediabetes	613 (10.7%)	1116 (14.8%)	1587 (18.3%)	
	No	4555 (79.5%)	5182 (68.5%)	4681 (53.9%)	
Heart failure	No	5685 (98.1%)	7526 (97.3%)	8647 (95.7%)	< 0.001
	Yes	108 (1.9%)	205 (2.7%)	388 (4.3%)	
Angina	No	5701 (98.6%)	7514 (97.3%)	8702 (96.3%)	< 0.001
	Yes	82 (1.4%)	209 (2.7%)	333 (3.7%)	
Heart attack	No	5629 (97.1%)	7410 (95.7%)	8562 (94.6%)	< 0.001
	Yes	167 (2.9%)	330 (4.3%)	492 (5.4%)	
Stroke	No	5644 (97.3%)	7459 (96.3%)	8669 (95.7%)	< 0.001
	Yes	154 (2.7%)	285 (3.7%)	389 (4.3%)	
Hypertension	No	4257 (73.4%)	5029 (64.9%)	5223 (57.6%)	< 0.001
	Yes	1546 (26.6%)	2719 (35.1%)	3844 (42.4%)	
Coronary heart disease	No	5404 (93.1%)	7011 (90.5%)	7941 (87.6%)	< 0.001
	Yes	399 (6.9%)	739 (9.5%)	1,127 (12.4%)	

*T1–T3: Tertile 1–3.*

In subgroup analysis, ethnicity modified the association of VAI with hypertension. The OR values for Non-Hispanic White participants were larger than those for other ethnicities, at 1.54 (95% CI, 1.33–1.79) for VAI tertile 2 and 2.14 (95% CI, 1.86–2.47) for tertile 3, relative to tertile 1 (Supplementary Table 2). In total, 8,687 participants had more than one CCD (Supplementary Table 3) and when compared with participants with no CCDs in the full model, the OR values for 1–3 CCDs across VAI score tertiles were 1.53 (95% CI, 1.27–1.85) and 2.26 (95% CI, 1.85–2.76) for tertiles 2 and 3, relative to tertile 1, respectively; while the OR values for more than 3 CCDs were 1.95 (95% CI, 1.35–2.82) and 3.06 (95% CI, 2.13–4.40) (Supplementary Table 4).

### The Dose-Response Relationship Between Visceral Adiposity Index Score and Cardiovascular and Cerebrovascular Diseases

**FIGURE 2:**
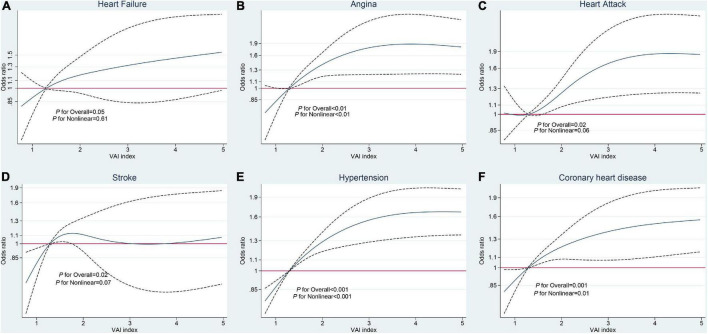
Restricted cubic spline analysis of the relationships between visceral adiposity index (VAI) and cardiovascular and cerebrovascular diseases (CCDs). **(A)** Heart failure. **(B)** Angina. **(C)** Heart attack. **(D)** Stroke. **(E)** Hypertension. **(F)** Coronary heart disease.

Restricted cubic spline regression was applied to explore non-linear associations between VAI scores and outcomes. We did not find any non-linear association between VAI and heart failure (overall *p* = 0.05, *p* for non-linear = 0.61) but did find a significant non-linear association between VAI score and angina (overall *p* = 0.03, *p* for non-linear = 0.03), with an initial accelerated curve when VAI score exceeded the median value, which began to decrease at a score of approximately 3. Analysis of the association between VAI and heart attack resulted in a significant overall *p*-value, while the non-linear association was marginally significant (overall *p* = 0.02, *p* for non-linear = 0.06), with similar results for the relationship between VAI and stroke (overall *p* = 0.03, *p* for non-linear = 0.07). Similarly, the relationship between VAI and hypertension followed a parabolic curve (overall *p* < 0.001, *p* for non-linear < 0.001), and a strong non-linear association was also found between VAI and coronary heart disease (overall *p* = 0.001, *p* for non-linear = 0.01; Figure 2).

### Sensitivity Analysis

For sensitivity analysis, VAI scores were categorized into quartiles and weighted multivariable logistic regression that were applied to investigate the associations between VAI and CCDs (Table 4). In the fully adjusted model, no significant association between VAI and heart failure was detected (*p* > 0.05). In contrast, higher VAI was associated with an increased risk of angina, with OR values of 1.33 (95% CI, 0.68–2.59), 2.18 (95% CI, 1.11–4.26), and 2.92 (95% CI, 1.49–5.69) for the second, third, and fourth VAI quartiles, respectively, relative to the first quartile. Further, the fourth quartile VAI score was associated with a 1.77-fold increased heart attack risk relative to the first quartile (95% CI, 1.09–2.88). However, the association between VAI score and risk of stroke was reduced and became non-significant, when the analysis of VAI score was changed from tertiles to quartiles (OR, 95% CI values: 1.34, 0.65–2.78; 1.68, 0.84–3.33; and 1.74, 0.92–3.28 in quartiles two, three, and four, relative to one, respectively). In addition, we detected a gradual increase in the risk for hypertension according to VAI quartiles, with corresponding OR, 95% CI values of 1.35, 1.10–1.66; 1.68, 1.40–2.00; and 2.37, 1.90–2.97 in quartiles two, three, and four, respectively. The strongest association of a CCD with VAI quartile score was with coronary heart disease (OR, 95% CI values: 1.14, 0.75–1.74; 1.45, 0.99–2.12; and 1.89, 1.24–2.86 for quartiles two, three, and four, relative to one, respectively; Table 3).

**TABLE 3:** Weighted logistic model for the association of VAI tertiles with CCDs in NHANES (1999–2018).

	VAI tertiles				
CCDs	T1	T2		T3	
**Heart Failure**					
Model 1	1.00	1.32	(0.96–1.80)	2.32	(1.67–3.21)
Model 2	1.00	1.67	(0.92–3.05)	2.27	(1.11–4.66)
Model 3	**1.00**	**1.57**	**(0.86–2.85)**	**1.89**	**(0.94–3.80)**
**Angina**					
Model 1	1.00	1.97	(1.37–2.83)	2.99	(2.15–4.16)
Model 2	1.00	1.89	(1.02–3.50)	3.25	(1.93–5.45)
Model 3	**1.00**	**1.80**	**(0.97–3.36)**	**2.86**	**(1.68–4.85)**
**Heart Attack**					
Model 1	1.00	1.47	(1.12–1.93)	1.92	(1.47–2.51)
Model 2	1.00	1.41	(0.90–2.21)	2.04	(1.33–3.13)
Model 3	**1.00**	**1.34**	**(0.86–2.10)**	**1.75**	**(1.14–2.69)**
**Stroke**					
Model 1	1.00	1.32	(0.98–1.78)	1.51	(1.19–1.91)
Model 2	1.00	1.87	(1.09–3.21)	2.30	(1.45–3.63)
Model 3	**1.00**	**1.78**	**(1.03–3.06)**	**2.01**	**(1.23–3.26)**
**Hypertension**					
Model 1	1.00	1.61	(1.44–1.80)	2.28	(2.04–2.54)
Model 2	1.00	1.63	(1.36–1.95)	2.54	(2.09–3.09)
Model 3	**1.00**	**1.56**	**(1.30–1.87)**	**2.28**	**(1.86–2.78)**
**Coronary heart disease**					
Model 1	1.00	1.28	(1.06–1.54)	1.84	(1.55–2.18)
Model 2	1.00	1.49	(1.12–1.99)	2.04	(1.51–2.74)
Model 3	**1.00**	**1.42**	**(1.06–1.92)**	**1.78**	**(1.32–2.41)**

*Model 1, adjusted for age and sex; Model 2, adjusted for age, sex, ethnicity, income, and education; Model 3, adjusted for age, sex, ethnicity, income, education, smoking, and diabetes. T1–T3: Tertile 1–3. The bold values indicates the estimation from the final model.*

**TABLE 4:** Weighted logistic model for the association of VAI quartiles with CCDs in NHANES (1999–2018).

	VAI quartile						
CCD	Q1	Q2		Q3		Q4	
Heart Failure	1	0.98	(0.48–1.99)	1.15	(0.56–2.39)	1.72	(0.79–3.72)
Angina	1	1.33	(0.68–2.59)	2.18	(1.11–4.26)	2.92	(1.49–5.69)
Heart attack	1	1.13	(0.69–1.87)	1.00	(0.62–1.61)	1.77	(1.09–2.88)
Stroke	1	1.34	(0.65–2.78)	1.68	(0.84–3.33)	1.74	(0.93–3.28)
Hypertension	1	1.35	(1.10–1.66)	1.68	(1.40–2.00)	2.37	(1.90–2.97)

*Adjusted for age, sex, ethnicity, income, education, smoking, and diabetes. Q1–Q4: Quartiles 1–4.*

## Discussion

In this study, we investigated the relationship between VAI and CCDs in a large sample of Americans and found that the highest VAI score classification was significantly associated with an increased risk for all CCDs tested, except for heart failure, and that the predictive power of VAI was better than other independently associated factors. In the analysis of the association of VAI with CCD comorbidity, a higher VAI index was associated with an increased risk of comorbid CCDs. In addition, non-linear relationships of VAI with angina, hypertension, and coronary heart disease were detected.

In our study, a high VAI score was associated with angina, stroke, hypertension, coronary heart disease, and heart attack. A few studies have demonstrated the heterogeneity of the predictive ability of VAI for different CCDs using data from large populations; however, previous studies have reported relationships between VAI and metabolic syndrome or cardiovascular risk factors. Throughout research about the relationship between metabolic disease and VAI, results from the analysis of the Chinese Health and Nutritional Survey that included 7,930 adults suggested that VAI was an independent indicator for diabetes (13). Further, statistics from the China H-Type Hypertension Registry Study of 8,591 adults showed that VAI was inversely associated with estimated glomerular filtration rate and the additional risk of chronic kidney disease (14). A cross-sectional study of 854 adults in Brazil indicated that the greater VAI score was associated with high-grade blood uric acid, leading to hyperuricemia (15). Some studies have made considerable efforts to analyze associations between VAI and cardiovascular risk factors, such as smoking, alcohol consumption, sedentary behavior, unhealthy diet, and age. An early NHANES study (2011–2014) supported the hypothesis that the VAI is associated with decreased “Dietary Approaches to Stop Hypertension” score, which can drastically reduce weight and lower the blood pressure in participants with hypertension (16, 17). In the period after quitting smoking, individual weight was significantly increased, but VAI score was reduced (18). Daily physical activity behaviors are essential indicators for CCDs. A cross-sectional study of 3,543 participants underscored that sedentary time was positively associated with VAI, while standing and walking times were negatively associated with VAI (19). Therefore, the VAI may cause CCDs *via* a series of markers, such as persistent unhealthy lifestyle and changes in blood biomarkers, and the index may be more sensitive and stable for examination of CCDs, due to its diverse parameters. In contrast, there was no significant association between VAI score and heart failure. Although obesity may raise the risk of heart failure onset, obesity has also been correlated with improved outcomes. This paradox may have influenced our estimates in a pooled cross-sectional study (20). Additionally, different obesity phenotypes may lead to different incidence rates, mortality rates, and outcomes of heart failure (20).

The predictive power of the VAI index was superior to each of the other independent factors analyzed in our study. Visceral adipose tissue (VAT) has previously been reported as a strong biomarker to predict CVDs. In the Framingham Heart Study, the VAT ratio had more predictive power for CVD risk among middle-aged women than BMI and WC (21). Additionally, in another study that involved 1,498 primary care patients, the research found that the *C* statistic of VAI for cardiovascular risk was significantly different from those of BMI, WC, and TG-to-HDL ratio, while for cerebrovascular risk, differences of VAI with BMI and WC were detected, but not with TG-to-HDL ratio (7). In a nested case-control study of a population of 1,052 Chinese individuals, the highest VAI quartile was strongly associated with an increased risk of coronary heart disease (22). Further, the VAI was associated with chronic inflammation and insulin resistance, due to increases in free fatty acids, interleukin-6, tumor necrosis factor-α, and decreased adiponectin production, related to VAT (23). Inflammatory cytokines and reactive oxygen species lead to decreased nitric oxide concentrations, which are associated with a higher risk of arterial stiffness (24–27). Additionally, large quantities of fatty acids, due to accumulative visceral adiposity and chronic inflammation, can promote the likelihood of insulin resistance, which is associated with atherosclerosis (28–31). Moreover, VAT is considered a sign of dysfunctional adipose tissue and ectopic fat deposition, leading to excess energy and subcutaneous adipose tissue storage (23).

Evaluation of dose-response relationships indicated that those of VAI with angina, coronary heart disease, and hypertension were parabolic (non-linear). Coronary heart disease onset is initiated by coronary artery arteriosclerosis, with angina as the main characteristic, suggesting myocardial ischemia and hypoxia (32). Atherosclerosis is always associated with hyperlipidemia; hence, VAI may be significantly increased due to the regulation of HDL (33). In addition, the arterial-wall tension arising from hypertension can lead to acceleration and deterioration of atherosclerosis of coronary and cerebral vessels. Moreover, hypertension may increase the susceptibility of small and large arteries to atherosclerosis. Therefore, individuals with hypertension can also suffer from atherosclerosis (34). In later stages, the non-linear curve presented a stable trend and maintained a high OR. This phenomenon might stem from the “obesity paradox,” which has been extensively debated in the context of stroke, myocardial infarction, and heart failure, where J-shaped curves between obesity and mortality have been observed in some longitudinal studies (35–39). In addition, the phenomenon of the “survivor paradox” should be considered (40), that is, participants with high VAI levels who suffered premature death due to genetic or environmental vulnerability were excluded from the study, while the survival of participants with high VAI may be attributable to various genetic variants, better medical care, and good hygiene.

This study has some strengths. First, we used ten cycles of NHANES data to represent the general population of the USA. Second, weighted estimation was adopted to overcome bias due to oversampling, and Taylor-linearized estimation was used to correct the actual variance. Third, extensive data on CVDs were investigated in this study, involving up to six subtypes, providing a comprehensive representation of the cardiovascular status of the American population. This study also has several limitations. Our results were driven by the observational study design, resulting in the challenges of demonstrating reverse causation and unobservable confounding. Further, CCD subtypes were determined based on questionnaire interview data; hence, disease severity could not be assessed.

In summary, the VAI score was positively associated with angina, heart attack, stroke, hypertension, and coronary heart disease but not heart failure, and the associations of the VAI score with angina, hypertension, and coronary heart disease were non-linear.

## Data Availability Statement

Publicly available datasets were analyzed in this study. This data can be found here: NHANES website (https://www.cdc.gov/nchs/nhanes/index.htm).

## Ethics Statement

The studies involving human participants were reviewed and approved by the NHANES was approved by National Centre for Health Statistics Institutional Ethics Review Board, and all the subjects agreed on the survey and completed the written consent. The patients/participants provided their written informed consent to participate in this study.

## Author Contributions

YZ and QH contributed to the conception, analysis, and interpretation of data, drafted the report, and managed the final version for publication. QH, WZ, SS, JY, and MY contributed to the analyzing and interpreting the data, commented on the report, revised the manuscript, and approved the final version for submission. All authors have read and agreed on the published version of the manuscript.

## Conflict of Interest

The authors declare that the research was conducted in the absence of any commercial or financial relationships that could be construed as a potential conflict of interest.

## Publisher’s Note

All claims expressed in this article are solely those of the authors and do not necessarily represent those of their affiliated organizations, or those of the publisher, the editors and the reviewers. Any product that may be evaluated in this article, or claim that may be made by its manufacturer, is not guaranteed or endorsed by the publisher.

## Footnotes

1
https://www.cdc.gov/nchs/nhanes/index.htm

## Abbreviations

AUC: area under the receiver-operating characteristic curve
BMI: body mass index
CCDs: cardiovascular and cerebrovascular diseases
CI: confidence interval
CT: computed tomography
CVD: cardiovascular disease
HDL: high-density lipoprotein
MRI: magnetic resonance imaging
NHANES: National Health and Nutrition Examination Survey
OR: odds ratio
ROC: receiver-operating characteristic
TG: triglycerides
USA: United States of America
VAI: visceral adiposity index
WC: waist circumference.
