# Supplementary material for: Non-linear Associations Between Visceral Adiposity Index and Cardiovascular and Cerebrovascular Diseases: Results From the NHANES (1999–2018)
Source: Front Cardiovasc Med. 2022 Jun 24;9:908020. doi: 10.3389/fcvm.2022.908020 (PMC9263190; doi:10.3389/fcvm.2022.908020)
Supplement: Supplementary file 1 [file Data_Sheet_1.docx]

***Supplementary files***

**Supplementary Table 1.** ROC curve analysis of prevalent CCDs according to VAI, TG, BMI, WC, and HDL.

| **CCD** | **ROC Area** | **SE** | **95% Confidence Interval** | |
| --- | --- | --- | --- | --- |
| **Heart failure*** |  |  |  | |
| VAI | **0.58** | 0.01 | 0.56 | 0.60 |
| TG | 0.54 | 0.01 | 0.52 | 0.56 |
| BMI | 0.56 | 0.01 | 0.54 | 0.58 |
| WC | 0.55 | 0.00 | 0.54 | 0.56 |
| HDL | 0.52 | 0.01 | 0.51 | 0.54 |
| **Angina*** |  |  |  |  |
| VAI | **0.58** | 0.01 | 0.56 | 0.60 |
| TG | 0.53 | 0.01 | 0.51 | 0.55 |
| BMI | 0.54 | 0.01 | 0.53 | 0.56 |
| WC | 0.55 | 0.00 | 0.54 | 0.56 |
| HDL | 0.53 | 0.01 | 0.51 | 0.55 |
| **Heart attack*** |  |  |  |  |
| VAI | **0.56** | 0.01 | 0.54 | 0.57 |
| TG | 0.52 | 0.01 | 0.51 | 0.54 |
| BMI | 0.52 | 0.01 | 0.51 | 0.54 |
| WC | 0.55 | 0.00 | 0.54 | 0.55 |
| HDL | 0.54 | 0.01 | 0.52 | 0.55 |
| **Stroke*** |  |  |  |  |
| VAI | **0.54** | 0.01 | 0.52 | 0.56 |
| TG | 0.51 | 0.01 | 0.49 | 0.52 |
| BMI | 0.51 | 0.01 | 0.50 | 0.53 |
| WC | 0.53 | 0.01 | 0.53 | 0.54 |
| HDL | 0.51 | 0.01 | 0.49 | 0.52 |
| **Hypertension*** |  |  |  |  |
| VAI | **0.54** | 0.01 | 0.52 | 0.56 |
| TG | 0.51 | 0.01 | 0.49 | 0.52 |
| BMI | 0.51 | 0.01 | 0.50 | 0.53 |
| WC | 0.53 | 0.01 | 0.53 | 0.54 |
| HDL | 0.51 | 0.01 | 0.49 | 0.52 |
| **Coronary heart disease*** |  |  |  |  |
| VAI | **0.56** | 0.01 | 0.55 | 0.57 |
| TG | 0.52 | 0.01 | 0.51 | 0.53 |
| BMI | 0.53 | 0.00 | 0.52 | 0.54 |
| WC | 0.55 | 0.00 | 0.54 | 0.55 |
| HDL | 0.53 | 0.00 | 0.52 | 0.53 |

VAI, Visceral adiposity index; TG, Triglycerides; BMI, Body mass index; WC, Waist circumference; HDL, high-density lipoprotein. *Significant difference between VAI and other factors (*P* < 0.001).

**Supplementary Table 2.** Subgroup analysis of associations between VAI and CCDs according to sex and ethnicity.

|  | **VAI tertile** | | | | |  |
| --- | --- | --- | --- | --- | --- | --- |
| **CCDs** | **T1** | **T2** | | **T3** | | ***P* for interaction** |
| **Heart failure** |  |  |  |  |  |  |
| **Ethnicity** |  |  |  |  |  | 0.96 |
| Mexican American | 1 | 1.44 | (0.52–3.99) | 1.64 | (0.63–4.24) |  |
| Non-Hispanic Black | 1 | 1.19 | (0.73–1.94) | 1.72 | (1.05–2.80) |  |
| Non-Hispanic White | 1 | 1.15 | (0.79–1.67) | 1.51 | (1.06–2.15) |  |
| Other | 1 | 0.83 | (0.38–1.80) | 1.35 | (0.67–2.72) |  |
| **Sex** |  |  |  |  |  | 0.56 |
| Female | 1 | 1.00 | (0.65–1.52) | 1.24 | (0.84–1.85) |  |
| Male | 1 | 1.22 | (0.87–1.72) | 1.71 | (1.24–2.36) |  |
| **Angina** |  |  |  |  |  |  |
| **Ethnicity** |  |  |  |  |  | 0.72 |
| Mexican American | 1 | 2.41 | (0.82–7.08) | 1.99 | (0.70–5.67) |  |
| Non-Hispanic Black | 1 | 1.34 | (0.74–2.42) | 1.31 | (0.69–2.50) |  |
| Non-Hispanic White | 1 | 1.37 | (0.94–2.01) | 1.74 | (1.21–2.49) |  |
| Other | 1 | 1.12 | (0.50–2.52) | 1.10 | (0.51–2.39) |  |
| **Sex** |  |  |  |  |  | 0.36 |
| Female | 1 | 1.92 | (1.15–3.20) | 2.01 | (1.23–3.31) |  |
| Male | 1 | 1.27 | (0.90–1.80) | 1.64 | (1.18–2.29) |  |
| **Heart attack** |  |  |  |  |  |  |
| **Ethnicity** |  |  |  |  |  | 0.85 |
| Mexican American | 1 | 1.01 | (0.43–2.34) | 1.49 | (0.69–3.21) |  |
| Non-Hispanic Black | 1 | 1.06 | (0.68–1.63) | 1.38 | (0.89–2.16) |  |
| Non-Hispanic White | 1 | 1.25 | (0.93–1.69) | 1.26 | (0.95–1.68) |  |
| Other | 1 | 1.15 | (0.61–2.19) | 1.08 | (0.58–1.99) |  |
| **Sex** |  |  |  |  |  | 0.48 |
| Female | 1 | 1.33 | (0.89–1.99) | 1.31 | (0.88–1.93) |  |
| Male | 1 | 1.19 | (0.91–1.55) | 1.48 | (1.15–1.90) |  |
| **Stroke** |  |  |  |  |  |  |
| **Ethnicity** |  |  |  |  |  | 0.19 |
| Mexican American | 1 | 0.72 | (0.36–1.46) | 0.80 | (0.42–1.51) |  |
| Non-Hispanic Black | 1 | 1.48 | (1.01–2.17) | 1.49 | (0.98–2.26) |  |
| Non-Hispanic White | 1 | 1.39 | (0.98–1.98) | 1.47 | (1.05–2.07) |  |
| Other | 1 | 0.98 | (0.53–1.81) | 0.74 | (0.40–1.37) |  |
| **Sex** |  |  |  |  |  | 0.15 |
| Female | 1 | 1.14 | (0.82–1.58) | 0.95 | (0.69–1.31) |  |
| Male | 1 | 1.21 | (0.89–1.65) | 1.35 | (1.00–1.82) |  |
| **Hypertension** |  |  |  |  |  |  |
| **Ethnicity** |  |  |  |  |  | **0.01** |
| Mexican American | 1 | 1.04 | (0.80–1.37) | 1.32 | (1.03–1.70) |  |
| Non-Hispanic Black | 1 | 1.24 | (1.03–1.49) | 1.53 | (1.24–1.90) |  |
| Non-Hispanic White | 1 | 1.54 | (1.33–1.79) | 2.14 | (1.86–2.47) |  |
| Other | 1 | 1.53 | (1.18–1.98) | 1.91 | (1.48–2.47) |  |
| **Sex** |  |  |  |  |  | 0.43 |
| Female | 1 | 1.26 | (1.09–1.45) | 1.48 | (1.28–1.70) |  |
| Male | 1 | 1.18 | (1.04–1.34) | 1.38 | (1.22–1.56) |  |
| **Coronary heart disease** | |  |  |  |  |  |
| **Ethnicity** |  |  |  |  |  | 0.69 |
| Mexican American | 1 | 0.94 | (0.57–1.55) | 1.25 | (0.80–1.98) |  |
| Non-Hispanic Black | 1 | 1.25 | (0.95–1.64) | 1.52 | (1.13–2.03) |  |
| Non-Hispanic White | 1 | 1.32 | (1.07–1.64) | 1.50 | (1.23–1.85) |  |
| Other | 1 | 1.10 | (0.72–1.67) | 1.11 | (0.74–1.66) |  |
| **Sex** |  |  |  |  |  | 0.39 |
| Female | 1 | 1.23 | (0.97–1.55) | 1.27 | (1.01–1.59) |  |
| Male | 1 | 1.22 | (1.01–1.49) | 1.56 | (1.30–1.88) |  |

**Supplementary Table 3.** Numbers of co-existing CCDs in NHANS participants (1999–2018).

| **Co-existing CCDs** | | **Number of participants** | **Percent** | | **Cumulative percent** | | |
| --- | --- | --- | --- | --- | --- | --- | --- |
| 0 | 13,831 | | | 61.14 | | 61.14 |  |
| 1 | 113 | | | 0.50 | | 61.64 |  |
| 2 | 6756 | | | 29.86 | | 91.50 |  |
| 3 | 430 | | | 1.90 | | 93.40 |  |
| 4 | 926 | | | 4.09 | | 97.50 |  |
| 5 | 416 | | | 1.84 | | 99.34 |  |
| 6 | 124 | | | 0.55 | | 99.89 |  |
| 7 | 26 | | | 0.11 | | 100 |  |
| Total | 22,622 | | | 100 | | 100 |  |

**Supplementary Table 4.** Weighted multinomial model of the association between VAI tertiles and co-existing CCDs in NHANS (1999–2018).

| **Co-existing CCDs** | **T1** | **T2** | **T3** |
| --- | --- | --- | --- |
|  | **95% Confidence Interval** | | |
| **Model 1** |  |  |  |
| No CCDs | Reference | Reference | Reference |
| 1–3 CCDs | 1 | 1.58 (1.42–1.77) | 2.23 (1.99–2.50) |
| > 3 CCDs | 1 | 1.84 (1.45–2.33) | 3.30 (2.61–4.18) |
| **Model 2** |  |  |  |
| No CCDs | Reference | Reference | Reference |
| 1–3 CCDs | 1 | 1.58 (1.31–1.93) | 2.47 (2.02–3.00) |
| > 3 CCDs | 1 | 2.12 (1.49–3.02) | 3.81 (2.65–5.47) |
| **Model 3** |  |  |  |
| No CCDs | Reference | Reference | Reference |
| 1–3 CCDs | 1 | 1.53 (1.27–1.85) | 2.26 (1.85–2.76) |
| > 3 CCDs | 1 | 1.95 (1.35–2.82) | 3.06 (2.13–4.40) |

T1, Tertile 1; T2, Tertile 2; T3, Tertile 3.
